# Identifying Audiences of E-Infrastructures - Tools for Measuring Impact

**DOI:** 10.1371/journal.pone.0050943

**Published:** 2012-12-11

**Authors:** Daphne Duin, David King, Peter van den Besselaar

**Affiliations:** 1 Department of Organization Sciences and Network Institute, VU-University Amsterdam, Amsterdam, The Netherlands; 2 Department of Computing, The Open University, Milton Keynes, United Kingdom; The Centre for Research and Technology, Hellas, Greece

## Abstract

Research evaluation should take into account the intended scholarly and non-scholarly audiences of the research output. This holds too for research infrastructures, which often aim at serving a large variety of audiences. With research and research infrastructures moving to the web, new possibilities are emerging for evaluation metrics. This paper proposes a feasible indicator for measuring the scope of audiences who use web-based e-infrastructures, as well as the frequency of use. In order to apply this indicator, a method is needed for classifying visitors to e-infrastructures into relevant user categories. The paper proposes such a method, based on an inductive logic program and a Bayesian classifier. The method is tested, showing that the visitors are efficiently classified with 90% accuracy into the selected categories. Consequently, the method can be used to evaluate the use of the e-infrastructure within and outside academia.

## Introduction

Much modern research is dependent on large research facilities and infrastructures. However, infrastructures are increasingly becoming *e-infrastructures* or *cyber-infrastructures*, and significant investments have been made over the last decade often supported by publicly funded e-infrastructure initiatives. E-infrastructures for research are facilities that provide researchers with networked access to digital data, collections and archives, to analytical (mainly computational) tools and computing power, and to collaboratories, tools for large scale and remote communication and collaboration. Doing research using these new infrastructures is often called e-science [1]–[2]. Several countries have specific programs and institutes for e-science or cyber-science, such as the US [3], the UK [4], and the Netherlands [5].

The rationale behind e-infrastructures is that moving research, output and communication to web-based systems facilitates the integration of distributed expertise and fragmented data, while improving access to these resources for scholars and for various interested audiences from wider society. Given the large investments in e-infrastructures, evaluating their impact is a new challenge for policy makers and for those researchers designing, developing and operating these infrastructures [1]. Crucial in every impact evaluation is the identification of different types of users (audiences) and use [6], and different e-infrastructure may have different target audience(s).

Web analytics packages, such as Google Analytics, can be used to generate information on the visitors (users) of web-based e-infrastructures, notably through identification of the names of the *visiting organizations* (VO): the organizations that are linked to the IP addresses stored in the sites’ log files. However, without any further data treatment the names of VOs have little meaning for evaluation purposes. This paper addresses the methodological question: *can we build an identification and classification method with computational techniques, in order to cluster Visiting Organizations to scientific websites into meaningful categories?* We present a computer-aided machine-learning approach and compare it with a manual approach in terms adequacy, efficiency, and robustness. The computational approach builds on a data filtering and clustering method for identifying organizations visiting e-infrastructures, combining an inductive logic program with a Bayesian classifier.

The rest of the paper is organized as follows. After a review of the research evaluation problems that motivated the development of the method, in the Materials and Methods section we introduce the case, data and methods used in our work. In the Results section, we give the outcomes of the classification tests. In the final section, we present the main findings and steps for future work. In the supporting information we discuss why the approach is useful for evaluating biodiversity research and research infrastructures (Text S1). We also provide additional details about the developed classification method (Text S2).

Our contribution aims to develop a method for an easy to use, intelligent tool to help e-infrastructure owners to study their audiences. This may help them to evaluate their facility in terms of the size and growth of the various audiences using it. The developed approach will be illustrated on a specific case.

### The Need for Alternative Metrics

Although the need for research evaluation metrics is generally acknowledged, the use of many established indicators is repeatedly criticized, focusing on four issues.

One main point of criticism is that the established citation-based indexes (e.g. Journal Impact Factor and the H-index) do not sufficiently take into account the differences in work and publication practices across disciplines, and may therefore inadequately measure output, impact and quality. This is most obvious in the cases of, but not limited to, social sciences and humanities [7], biodiversity research [8]–[9], and technical sciences [10].

A second criticism of citation-based evaluation metrics is that they only focus on one audience, peers, and therefore are one-dimensional. They account at best for the role of scientific output within the researchers’ discipline, but do not evaluate the impact the work might have on society at large [11]. Increasingly, policy makers and scholars emphasize that the assessment of scientific information and the science system should also account for its contributions outside academia [6], [12]–[14].

A third criticism is that the citation-based metrics only take into account a specific form of communicating research output, scholarly articles. They neglect the new forms of communication that have emerged with the advent of e-infrastructures [15], where case studies suggest the communication of research can have a large impact [16]. E-infrastructures offer new ways to communicate research findings to new audiences and to make additional scientific output, such as raw data and workflow development, more easily accessible outside the traditional publication route [17]. Consequently, e-infrastructures can enable the identification of users and their use of the research output outside academia [15], [18], [19].

A fourth criticism of the established metrics refers to the nature of the evaluation. Two types of evaluations can be distinguished, summative and formative [20]. Summative evaluation is meant to answer accountability questions, and often leads to ranking performance in comparison to others. However, increasingly it has been emphasized that evaluation should lead to learning and improving, in order to position oneself adequately: formative evaluation [6], [11]. Whereas external evaluators generally do summative evaluation, formative evaluation can be a powerful tool for the evaluated. The latter, however, requires a metric that can be easily deployed by researchers and research organizations to meet their own needs. New research performance metrics should therefore be easy to use, and not be time and resource consuming activities.

When taking these issues together, it becomes clear that in many cases “traditional” evaluation indicators do not account for diversity of use of scholarly output. Therefore indicators are needed, which fit the context where they are deployed, and which can be adjusted accordingly by its users. A core element of such metrics is the identification of the different addressed audiences, scholarly and societal. As most research output is currently on the web, identifying heterogeneous audiences is crucial for measuring impact in a multidimensional way. In other words, identifying categories of Visiting Organizations to the relevant websites, and measuring their size, growth, and intensity of use, would make a good indicator for evaluating parts of the work carried out by researchers. Such a metric accounts for more types of research output than only publications – such as data sets and analytical tools. And, the indicator helps identifying other users than peers only, and therefore covers the broader impact within *and* outside academia.

### Audience Research for e-science

As said, it is increasingly important for public sector services, like academia, to be able to demonstrate that they are used and valued by an appropriate audience. Here the web offers possibilities. Web audience research can be applied to study *who* is using a web services and *how* they use it. Answering these questions should help, among others, to demonstrate accountability to funders, support IT development or to evaluate the service in terms of reach to target audience.

A range of methodologies is available to study web services like e-infrastructures. There are quantitative methods, which make use of web server logs (logs stored when web browsers request a page from the web server). Previous studies investigated audience search behavior in resources like digital archives [21] scientific literature databases [22], mainly focusing on how websites are searched by visitors. Other quantitative methods are standard web analytics packages that generate reports with overall statistics about traffic and visitors to (see Fang [23] for an example of studying library websites) and user surveys inquiring background information from visitors to the web resources.

Qualitative methods also exist for studying science on the web such as focus group interviews [24], collecting feedback via help support forum, listservs or via a ‘contact us’ button on the website [25]. In addition, link analysis of inlinks can say something about the organizations or individuals that connect to the resource under study [15], [18]. Inlinks are “at an abstract level an endorsement of the target page by the author of the source page” [18 p23]. Except for the web analytics package all the other methods require analytical, technical or social research skills and so make web audience research a task for experts.

The purpose of our work is to develop a method to help identify the audiences coming to websites of researchers. We propose a method that (i) requires no specific skills of the site owner, and (ii) is capable of generating the information in little time and with little or no human input. Taking this into account, the web analytics tool Google Analytics [26] is a good starting point. Google Analytics is a free service that is simply activated by inserting code into each web page to be tracked. One of the features in the web report is the names of the visiting organizations (VOs) coming to the website, based on their internet service provider numbers. Often these lists with VOs’ names are rather obscure and hard to read. The method discussed in this paper proposes to cluster these VOs into meaningful categories, in order to help site maintainers to understand and to demonstrate the scope of the audience using their web resource. This will be helpful for accounting purposes or to evaluate if the site is reaching its target audience. We propose a computational classifier technique to cluster the VO names using a Bayesian classifier.

### Web Visiting Organizations

We suggest that the name of visiting organizations (VO) to an e-infrastructure will provide us with relevant characteristics of the users, including their expertise and organizational context. Through the identity of the VOs, we can learn about the audiences of research sites and infrastructures.

Some VOs are commercial internet service providers (ISP). These ISPs, mainly telecom and cable companies, provide access from peoples’ home, or from mobile devices. On the other hand, many companies, universities and government agencies, and non–governmental organizations act as an ‘ISP’ for their employees or membership. Through the name of the VO, (e.g ‘Vrije Universiteit Amsterdam’) we may be able to identify the nature and activities of the users. When connecting to the Internet through a computer network of the organization, web analytics packages (or the systems’ log files) will pick up the name and add it to their ‘visitors report’. Hence, web reports contain two types of VOs: those linked to the ‘general names’ of commercial ISPs and also to ‘specific names’ of a visitors’ organization. In the first case the name of the ISP does not tell us much about the affiliation of the user, in the second case the name will give a good indication of the users’ affiliation.

Therefore, this paper addresses two tasks: 1) to identify the different VOs visiting a research website; 2) to classify those VOs into relevant user categories. In this paper, we focus on methods to perform those tasks in a ‘doable’ way that does not require specialist expertise.

While a detailed consideration of relevant user categories depends on the context of the resource being evaluated, a broader categorization is still possible and often one may want to distinguish (i) the sector of use (e.g. peers, researchers in other fields, professionals, policy makers (government), industry, the general public); (ii) the subsector of use (e.g. within education: secondary education, higher education; within government: local, regional, national, supra national); and (iii) the thematic focus of the knowledge users (e.g. water management, biofuels).

## Materials and Methods

### The case

As all four points of critique on traditional evaluation metrics apply to biodiversity research, we used data from this field (see for more details Text S1), and chose the e-infrastructure Scratchpads for our study. Scratchpads are online platforms for collaborative work meeting the specific requirements of data sharing and collaborative analysis in biodiversity research. The Scratchpads platform is developed and maintained by a small team of developers at the Natural History Museum, London, and is built on the Drupal Content Management System. At the time of writing, the Scratchpads platform hosts more than 300 research collaboratories and has a global user community of more than 5,000 registered users. The individual sites (collaboratories) are maintained and managed by their owners, generally researchers in the specialty, and not by the Natural History Museum. Scratchpads allow geographically scattered specialists to collaborate, share and analyze data online. The scholarly use of Scratchpads ranges from blog type discussions to analyzing data sets and collaboratively writing scientific papers [27], [28]. Scratchpads owners can choose to what extent they make the content of their website publicly available. Scratchpads can be made available to people outside the academic biodiversity research community, such as NGOs, policy makers, and companies with an interest in biodiversity information, as well as the general public.

### Data

The VO data used in this study has been collected by Google Analytics, which provides statistical information about visits to web pages. The majority of Scratchpads (>90%) are hosted on the server of the Natural History Museum, London (NHM) under the domain name ‘myspecies.info’. In our study, we used Google Analytics reports on NHM server activity.

We chose to use Google Analytics in preference to other sources of data because it is relatively easy to make the reports available to Scratchpads owners. It is certainly easier than giving them access to the NHM’s server logs, which also contain visitor data. Having access to the Google Analytics reports allows Scratchpads owners to adjust the measurement tool we develop in this paper according to their own needs. A potential weakness of this approach is the reliance of Google Analytics on users permitting cookies in their web browser. Although visitors to Scratchpads have the option to turn off browser cookies, we have evidence from a survey that for the visitors included in this study this is not the case.

We used two datasets in our analysis, covering almost all Scratchpads: the first set is called our ‘initial data’, and the second set is our ‘gold standard test data’. We call the second set the ‘gold standard’ because it has been manually checked and reviewed by several independent researchers, hence it can be considered as 100% correct.

The initial data was taken from the period October 1, 2010 to March 31, 2011. At that time, there were just over 200 Scratchpads web sites. The data contains 16,484 unique VOs. It was used to develop our filter and classifying routines.

The second data set was taken from May 2011 (one month). This data set contains 6,728 unique VOs. We took the 1,000 VOs with the most visits to Scratchpads and hand marked them as either to include or exclude from the classifying routines. Thus, we had a test data set to assess the accuracy of the routines we created based on the ‘initial data’.

An important aspect of this type of data is its ‘long tail’. In the test data set of 1,000 VOs, there are 1,576 unique terms in the names of the VOs. The most frequent term is ‘of’ with 126 occurrences, the next most frequent is ‘university’ with 118 occurrences, then ‘de’ with 71 and ‘network’ with 60. This distribution does not follow an obvious pattern such as Zipf's Law. If we control for languages and aggregate variants of ‘of (‘de’, ‘do’, ‘du’, ‘of’ and ‘van’), we get 201 occurrences for this most frequent term set, followed by variants of ‘university’ at 179 occurrences as the next most frequent term set. This still does not seem to follow any known pattern. The presence of so many unique terms presents us with a challenge in categorizing the VOs, as it is difficult to identify meaningful patterns.

### Method

We test the reliability of two approaches to classifying VOs. One approach is purely manual; the other is computer-assisted. We use Google Analytics visitor information for Scratchpads as our test data.

To classify VOs in a meaningful way, we use a two-stage approach. The first stage is to filter out general VOs who are mostly commercial internet access providers and telecom companies. These cannot be classified meaningfully in terms of audiences. In the second stage, the remaining VOs are classified.

For the initial data set, a filter set was compiled manually. It consists of 173 terms that if found in the VO name the VO would be included for future analysis, and 8 terms that if found would exclude the VO, where include takes precedence over exclude. The filter removed the general VOs, and produced a relevant set of VOs for identification of audiences. The first task was to validate the manually derived filter set: can it be applied generally on other data sets. To do this, a gold standard test data set was created. To enhance the validation, a second filter set was compiled to compare results. Rather than create another manually, albeit independently, derived filter set for the comparison, a machine learner was used (the inductive logic program *aleph*), to look for patterns in and to induce rules from the marked data.

Aleph is a flexible program, in that it can identify statistically significant terms in the data both as complete terms and as templates. For example, ‘university’ and its language variants ‘universidad’, ‘universidade’, ‘universita’, ‘universitaet’, ‘universite’, ‘universiteit’ and ‘universitet’ are identified as good markers of VOs to be kept for use in the classification stage, and that these terms can be replaced by the template ‘universi’. Hence, the filter is easier for a human to read, and quicker for a computer to apply, because it contains only the template ‘universi’ instead of the eight different words used for ‘university’ in the data.

In summary, we have two data sets, our initial data set for development work, and a test data set for evaluation, and two contrasting filter sets to reduce the data sets to a manageable size for classification.

## Results

In this section, we discuss the results of clustering VOs in categories as indicated in section 1.2. We start from the idea that this is possible when the general VOs are removed from the list (1) and when we have a classifier that can group the VOs based on their names (2). We aim for a data treatment that will generate a high precision and recall. This data treatment could be done manually or with help of a computational technique. We compare the robustness of both approaches. In the last section, we discuss the weighing of (3) the number of ‘general VOs’ versus ‘specific VOs’ in the data set and how this enhances our understanding of the meaning of these results.

### Filtering: Reducing the Data for Classification

The first stage is to reduce the size of the data to manageable proportions for classifying the VOs. The results of applying the two filters sets are shown in Table 1. This first test shows a high degree of precision, suggesting that the methodology successfully identifies meaningful patterns in the VO names, and that these patterns can be used to filter the data.

**Table 1:** Comparison of include results for the two early filter sets on gold standard test data.

	6 term filter set	181 term filter set
precision:	0.98	0.92
recall:	0.73	0.97
f-measure:	0.84	0.94

The results not only helped improve the choice of keywords, but also suggested how to improve the filtering process. The process of refining the filter keywords is covered in Text S2. It became apparent during this testing that one needs to distinguish between terms and templates. A term matches only on a full word in the VO name, a template matches on that sequence of letters, even if those letters are part of another word in the VO name. The term filter proved particularly useful in eliminating false positives. For example, ‘cri’ was originally identified to match the acronym used by several French research centres, “*Centre de Recherches Interdisciplinaires”*, but also matched any VO that includes the word ‘subscribers’ in his name. By using ‘cri’ as a term solves this problem. To provide this functionality the filtering script was extended with strong exclude templates – a VO containing this pattern *must* be marked as *exclude*. It now includes the following hierarchy for identification of VOs:

- strong exclude terms – a VO containing this word **must** be marked as *exclude*
- include templates – a VO containing this pattern is marked as *include*
- include terms – a VO containing this word is marked as *include*
- exclude numbers – a VO containing a number is marked as *exclude*
- exclude templates – a VO containing this pattern is marked as *exclude*
- exclude terms – a VO containing this word is marked as *exclude*
- other terms – any remaining unmarked VOs are marked as *other.*

Although the script has become more sophisticated, we have kept the script relatively simple to use. The Scratchpads owner only has to provide a list of keywords in one of the categories listed above. All the complexities and the details of the use of regular expressions to apply these keywords to the VO names is hidden from the user.

Following the changes to the filter processing script, we consistently achieve precision above 0.95 when identifying VOs to *include* and to *exclude* for classifying. This performance comes at the cost of declining recall, though we do maintain a recall above 0.80. The underlying problem is the nature of our data, with its large number of unique terms. This problem is addressed through the ease of manual review of our results and the comprehensive log of the filtering process, which allows the user us to quickly identify VOs, even when working with new data that contains previously unseen VOs.

### Classifying: Identify Categories in the Data

Having reduced the data to a manageable size, we apply the three tiers of classification as an indicator for the use of Scratchpads [15]. For example, we distinguish in the first tier the following categories of VOs: Research & Education, Government, Industry, Media & Arts.

Some categories are easily made up from words in the name of the full VO such as “university” or “research” and can be grouped under the tier one category “research&education”. However, this approach is limited. For example, simply categorizing all VOs with the terms “health” or “medic*” in their names as “public health” meant that a range of research, educational, governmental and corporate affiliated VOs are wrongly categorized. Hence our adoption of a classifier tool to categorize the VOs to overcome this limitation. Given the relative sophistication of the filtering, our initial choice was to use a simple classifier to prove the validity of our approach. Our simple classifier was:

- *xnaïve*
- This means that all of the words in the VOs’ names are of equal value. Hence “university”, which is a strong indicator for “research&education” has the same value in classifying a VO as “research”, which covers “research&education” as well as other categories such as “agriculture/animal health” or “biodiversity/environment”.
- *without thresholds*
- Therefore all categories are of equal value. Hence, our classifier differs from used as a spam filter because that filter will fail to classify some documents because it cannot positively categorize the document as spam or clean. In contrast, because we have already filtered our ISP list to remove all those we want to exclude and any that are unknown, we expect to be able to classify all the remaining VOs. Therefore we have no threshold for the confidence of a classification, we simply apply the best match we can.
- *without other cues*
- Classifiers can incorporate custom rules specific to the domain, for example, we could make use of relationships across the three levels of classification to.

Our initial classification results are presented in Table 2. The results show a simple match and mismatch between the classifier’s classification of a VO and the manually marked classification. Table 2 demonstrates the effects of the different levels of abstraction in the three classification levels. Tier one, which addresses the VOs’ “sector” such as “research&education” or “government” is handled well even by this simple classifier. As the degree of abstraction increases, however, the accuracy of classification decreases. Attempting to classify tier three, the VOs’ “focus” leads to 49% accuracy. This is not unexpected given the simplicity of the implemented classifier. However, the log of the classifier shows the classification trigger values, which can be used to review the classifier output with Excel, leading to enhancing its accuracy (see for more information Text S2).

**Table 2:** Results using a simple classifier to apply all three tiers to the gold standard test data set.

	match	mismatch	accuracy
tier one (sector of use)	273	29	90%
tier two (subsector)	266	36	88%
tier three (thematic focus)	149	153	49%

Our results indicate that a simple classifier is not sufficient to categorize the VOs. However, we have relatively simple data, consisting solely of short VO names, and we are not looking for complex relationships. Therefore, a Bayesian classifier may be sufficient for our needs. A Bayesian classifier takes into account conditional probabilities that can be refined as more data is acquired. Firstly, Bayesian classifiers can be trained on small data sets, and as our tool is also to be used by the owners of individual sites (such as the Scratchpads of our case), they may well be working with small data sets. Secondly, a Bayesian classifier does not suffer from any of the three shortcomings identified in a simple classifier. For example, we found it necessary to weight the value of some terms more than others: if all terms carry equal weight then “institute” skews the classification to “chemistry” as chemists seem to favor working in institutes compared to working in departments or faculties. In our gold standard test data, this led to three incorrect classifications. However, weighting “institute” lower than “marine” corrects two of the false classifications, so that the “institute of marine biology of crete” and the “flanders marine institute” are now correctly classified as “biodiversity/environment”. Application of weighted terms in a Bayesian classifier achieved an immediate improvement in performance as shown in Table 3.

**Table 3:** Results using a Bayesian classifier with weighted terms to apply all three tiers to the gold standard test data set.

	match	mismatch	Accuracy
tier one	288	14	95%
tier two	278	24	92%
tier three	251	51	83%

The results indicate that we have a process that quickly produces usefully accurate classifications at tier one and tier two. We also have an acceptable level of classification at tier three. While more accurate results potentially could be obtained through using alternative classification techniques such as neural networks and support vector machines this would come at the cost of requiring greater computing power, which might not be available to Scratchpads owners, and carries the risk of over fitting, providing too many false classifications which would make the results less useful to the Scratchpads owners.

### Testing the Process

The method was tested on the total set of Scratchpads sites (myspecies.info, 341 sites) for the period of 1 May 2012 to 31 May 2012 and, and these results are compared with those of two individual sites over the same period. We filtered the data (as described above) and applied the classifier at the level of tier one to study the audience coming to the sites in the categories ‘Research & Education’; ‘Government’; ‘Industry’; ‘Media & Arts’.

**Figure 1:**
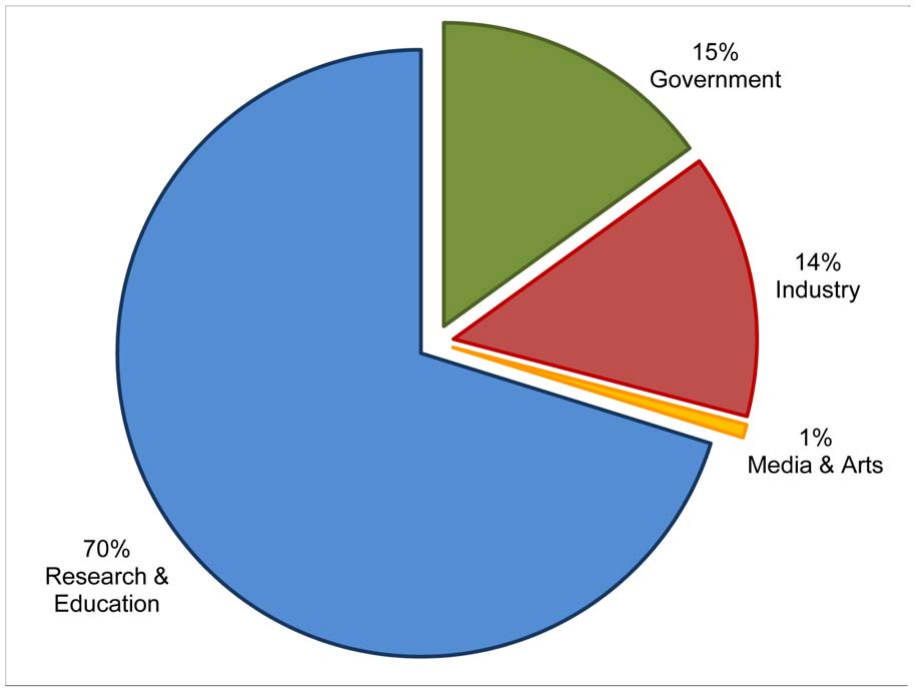
Visiting Organizations to Scratchpad domain *myspecies.info.* Based on Google Analytics visitor report on the domain of 341 Scratchpad sites over the period 1–31 May 2012. Visiting Organizations in chart represent 26% of the total number of Internet Service Providers (8263) that visited *myspecies.info* in that period and correspond to 76% of the total time spent on the sites.

**Figure 2:**
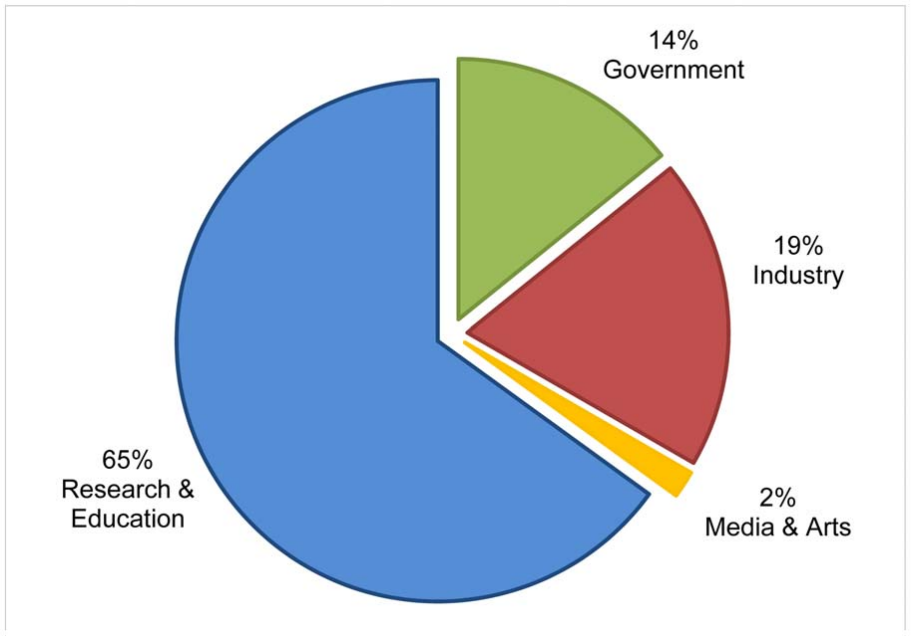
Visiting Organizations to Scratchpad *walkinginsect.inf*o. Based on Google Analytics visitor report to *walkinginsect.info* (fictional name) over the period 1–31 May 2012. Visiting Organizations in chart represent 16% of the total number of Internet Service Providers (749) that visited *walkinginsect.info* and correspond to 35% of the total time spent on the site.

**Figure 3:**
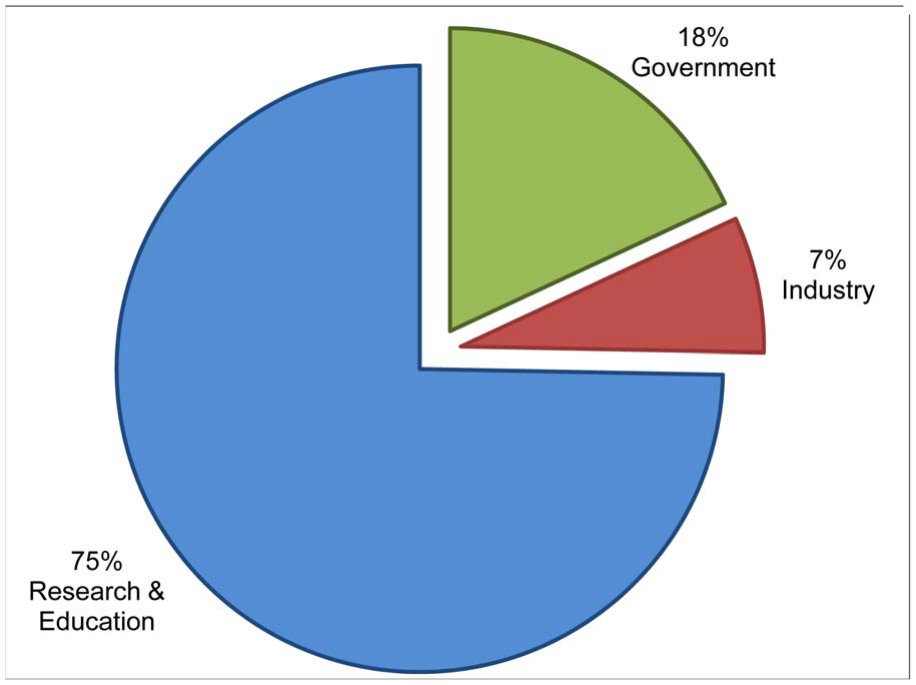
Visiting Organizations to Scratchpad *flyinginsect.info.* Based on Google Analytics visitors report to *flyinginsect.info* (fictional name) over the period 1–31 May 2012. Visiting Organizations in chart represent 28% of the total number of Internet Service Providers (294) to *flyinginsect.info* and correspond to 95% of the total time spent on the site.

The two individual sites were selected for the different scope of their audiences. Site one – with the fictional name *walkingsinsect.info -* is an example of a site of general interest covering both scientific research into the insects and also the hobbyists enjoyment of keeping and caring for the insects at home (Fig.2) It is expected to attract a wide audience, a mixture of academics, government organizations, nature lovers, etc. The second site – with the fictional name *flyinginsect.info* - is an example of a specialized site, that we expect would attract a mainly specialist audience of academics (Fig.3). These expectations are supported when comparing the number of sites filtered out in stage one of our method. For the whole domain (Fig.1) the classification is based on 26% of the total number of VOs; for the walkinginsect site 16%; for the flyinginsect site 28%. This difference could be caused by visits of hobbyist to the walkinginsect site, which are a group of users that are likely to be using a commercial ISP and therefore cannot be categorized with our classifier. However, for the actual time spent on the walkinginsect site, we found the VOs we could include in the classification stage accounted for 35% of the time spent on the site, suggesting that the included VOs represent professional (heavy) users. For the specialist flyinginsect site the figures are even more marked with the included VOs accounting for 95% of the time spent on site, and the excluded VOs 5%. Figure 1–3 shows the resulting classification of the different VOs for the domain and the two individual sites.

The domain including all sites (Fig. 1) takes a middle position regarding the percentage of VOs from the research&education sector. When comparing with the two unique sites, we expected to find different distributions of the VOs over the categories – which indeed is the case (Fig. 2 and 3). More specifically, we expected to have a much smaller share of the research&education sector in the ‘general’ walkinginsect site than in the researchers oriented flyinginsect site. But even in the walkinginsect site, research&education is still by large the biggest category. Interestingly, the site we expected to have the most homogenous audience (flyinginsect), attracted besides a large percentage of research&educational VOs also quite a number of governmental VOs.

In short, applying our classifier on different websites demonstrates that we are able to visualize the relative size of VOs by sector, enabling the evaluation and comparison of users. The results indicate that the chosen approach classifies VO data to meet the immediate needs of Scratchpads owners, and that we can invest in the development of a tool to make the classifier available for their use.

## Discussion

In this paper we argue that a set of relevant, sound and simple indicators for the evaluation of e-science infrastructures can be based on a measurement of the *types of scholarly and non-scholarly audiences* that are using the information provided by the e-infrastructure. Clustering the VOs visiting the sites, in our study Scratchpads, into meaningful user categories provides a valuable enhancement to plain web visits reports. Taking into account the number of visits and time spent on site provides additional information increasing the robustness of our method.

The resulting indicator sets are:

- three distributions (sectoral, subsectoral, thematical) of audiences, indicating which audiences are using the site
- number of visits weighted distributions of audiences, indicating the intensity of use
- time weighted distributions of audiences, indicating the depth of use, preferably measured over various timeslots, in order to observe change.

These *scope of audience* indicators follow the criteria for performance indicators as listed by Jacobs et al. [29], which are *relevance*, *availability and practicality, allowing for comparison, and utilization.* Firstly the indicator fulfills the requirement of ‘relevancy’ as it deals with aspects of the researchers’ job that are found important. The clustering of the audiences to Scratchpads is found important as it covers academic work and output in a broad sense. It is also relevant because it allows measuring the impact of the scholarly work outside science: its potential societal impact. Secondly, the indicator fits the requirement of being ‘available and practical’. The web statistics can be collected without interfering with the work and can be downloaded by every Scratchpads owner with basic computer skills. Third, the data allow for making comparisons, e.g. changes in the size of the different audiences over time. Fourth, knowing the audiences that use Scratchpads is useful for Scratchpads owners, as it helps answering questions as “do we reach our target audiences?” and “who uses our facility?”. For other users, the scope of audience indicator could serve as a social navigation tool (users that used this information also used…). Finally, researchers that manage a research site may use it for communicating the growing impact of their online work to, for example, their organizations and to funding bodies.

### Main Findings with Respect to the Process

The first data treatment aimed at removing the general VOs. We tested two filters and found that computer-aided filtering reached a higher precision than the manual developed filter (98% vs 92%). For the recall initially this was the other way around (73% vs 97%) though the result for the computer aided filter is robust enough for use. The computer-aided filter was improved through subsequent testing and more data. This means that with customized but relatively easy to use tools we were able to partly automate a filtering procedure and generate satisfying results. This is promising as an automated filter has many advantages over a purely manual approach. First, because selecting manually filter terms is very labor intensive, second, humans make irregular mistakes that are hard to find and remove. Moreover improving the machine learning filter is much more efficient than a manual filter, easier to reproduce and to reuse on other data.

The second data treatment developed on the ‘include’ keywords to cluster the VOs in meaningful categories for evaluation purposes. Examples of such categories are “Research & Education”, in which for example universities and science museums are classified, or “Government” in which government departments, municipalities, and research councils are classified. The classification results show that within a couple of minutes of downloading the data from Google Analytics, by running two scripts, we can classify with 90% accuracy the “sector” of the ISP. This gives a direct method for understanding the nature of Scratchpads’ audiences – and through repetition understanding the change that takes place over time.

After having classified the VOs, additional information was taken into account about the number of visits of each VO, and time each VO has spent on a Scratchpads site. This enables weighting: we do not only measure the distribution of audiences, but also the distribution of activity of use: although the ‘specific ISPs’ might represent only 16% of the VOs visiting generalist Scratchpads, they can account for about a third of the active use of that Scratchpads website. For specialist Scratchpads, this figure increases to 95% of the active use.

### Next Steps

Although the first findings are promising, we identified some additional work to incorporate into the development of the indicator as a tool for general use.

While the deployed data fulfills the requirements formulated by Jacobs et al. [29], nevertheless it has its limitations. More precisely, only visitors that are affiliated to specific VOs and use the institutional access are included in the classification analysis. In our sample, two thirds of the visits used general ISPs (e.g. Vodaphone, T-mobile) and cannot be classified. So firstly, the current method misses a considerable part of the user audiences. To overcome this limitation one could combine the use of the filter with a traditional evaluation method, such as an annual user survey which asks users to tick the box of the category they belong to. The results of this survey could be used to re-weight the findings of the method developed in this paper.

Secondly, the robustness of the first stage filtering can be further improved by adding a threshold to the ISPs visiting Scratchpads, for instance by only including those ISPs that stay on the site more than a minimum number of seconds or minutes. The precision of the second level classification can be further improved by using an ISPs’ location. For example, words such as “college” refer to a different level of education in different countries, and so can inform our subsector classification, which distinguishes between primary, secondary and tertiary education.

Thirdly, several possibilities exist to improve the overall accuracy of our tools. One possibility is the ability to vary the influence of each term when classifying ISPs. Similarly we can vary the importance of each category by setting different thresholds to be passed before an ISP is placed in a category. There are extra cues we can in the data we can incorporate, such as linking across the three tiers of classification. However, all of these possibilities need to be tested carefully, as our data has a long tail of unique terms. This may easily lead to many false positives being generated by inappropriate filters and classifications.

Improving the tools may partly be done through known techniques, such as Bayesian probability to weight individual words within an ISPs name. Another approach may be the application of empirical results for setting thresholds for our categories. Finally, it is useful to make further use of data mining techniques to look for and apply hidden patterns, such as the “of before of”.

An aspect of aleph is its ability to identify patterns in the data: it does not treat the data a simple bag of words, as does Google Analytics. Hence, in our work a VO in which ‘of’ precedes ‘of’ is one to include in the second stage classification. An example from our data is the ‘institute of marine biology of crete’, which is correctly recognized as a VO to include. This is one advantage of using a machine learner to look for patterns, as it is extremely unlikely that manual marking would have identified this significant pattern. Equally, such patterns may not be intuitive to the Scratchpads owners when they come to use the tool. Therefore, in the short-term interests of progressing our research we discarded pattern based rules so that our filters contain one word terms or templates only. However, we note this form of pattern-based rule may be a fruitful line of future research.

### Conclusion

Our contribution in this paper can be summarized in that we applied the concept of ‘audiences’ as an indicator for measuring research impact and we proposed a computational, adjustable and easy to use method to classify VOs into meaningful categories. The developed indicator follows the requirements for performance measures, such as relevance, availability and practicality, allowing for comparison, and utilization. Our data analysis demonstrates the ‘availability and practicality’ of the data as the basis of the metric.

The findings emphasize the value of computational techniques for data marking over human data marking. Most importantly, the classifier showed that that within minutes of downloading the data from Google Analytics, we could classify with 90% accuracy the “sector” of the VO (the first tier). This gives an immediate benefit to understanding the nature of who is accessing Scratchpads. Further improvements of the filtering and classifier are given and aim to support the development of an evaluation tool for individual researchers working with e-infrastructures like Scratchpads.

This study was first of all motivated by the problem that citation-based indexes do not take sufficient account of the differences in work and publication practices of various scientific disciplines and work settings. A second motivation for our work was to propose a metric that takes into account user categories outside academia. A third reason for our work is the move of science to the web, and therefore to develop a metric that fits e-science. The fourth and final incentive for our work was to develop a metric in line with what Van Raan [30] wrote about measuring science: “we need evaluation instruments that serve scientist as a grateful user, instead of an instrument as a vulnerable target (p.26)” and so propose a formative metric in contrast to a summative metric.

Summarizing, research output and its audiences are heterogeneous. Therefore, there is a need for tools to identify and measure these audiences, in order to enable relevant research evaluation. This paper demonstrates how this task can be accomplished.

## Supporting Information

Text S1 **Evaluation metrics for biodiversity.** A more detailed discussion of issues in research evaluation of biodiversity and taxonomy research
(DOCX)

Text S2 **Development of the two filter sets.** This describes in more detail the development of the two filter sets.
(DOCX)
