# Supplementary material for: Identifying Audiences of E-Infrastructures - Tools for Measuring Impact
Source: PLoS One. 2012 Dec 11;7(12):e50943. doi: 10.1371/journal.pone.0050943 (PMC3519820; doi:10.1371/journal.pone.0050943)
Supplement: Text S1 — Evaluation metrics for biodiversity. A more detailed discussion of issues in research evaluation of biodiversity and taxonomy research (DOCX) [file pone.0050943.s001.docx]

*Supplementary information S1.*

*Evaluation metrics in Biodiversity research*

Here we discuss the issues specific to the field of biodiversity and the need for an alternative metric to evaluate research performance. Over the last ten years or so, researchers in biodiversity sciences, covering the sub fields taxonomy and natural history sciences, have waved a red flag to policy makers and founding bodies for a too rigid use of the Web of Science citation measures which are, according to the scholars, not suitable to measure the quality of their work [1, 2-6]. Taxonomic and natural history journals generally have very low impact factors that disproportionally disfavor the research in comparison to other fields. Causes for this effect are multiple. Firstly, there is the convention that it is “considered unnecessary to cite original taxonomic descriptions or subsequent taxonomic revisions—the hypotheses behind species names—even when those hypotheses crucially impact a given study and its design” [3, p56]. Secondly, the field shows a high level of specialization around specific organisms. “Therefore the chance to become cited by colleagues is relatively rare compared with other fields” [2, p957]. Thirdly, as far as the original publications are cited, this falls outside the citation window that is taken into consideration in calculating the Journal Impact Factor – resulting in generally low impact factors for biodiversity journals. Lastly, some critics even take it further and argue that in biodiversity research, citations in general are a bad measure for quality because it are mainly the controversial species description that attract citations [7] and “original descriptions have to be referred to fore ever independent of the paper’s quality” [2, p957].

It is often stressed that society at large has a great interest in linking the data of the biodiversity communities in order to build the larger picture of knowledge on global biodiversity. This picture is important for scientific fields such as ecology, environmental studies, public health, biodiversity conservation and urban planning, and also for policy makers, societal stakeholders and the general public [8, 9]. An alternative metric that is able to demonstrate this impact would help making the information more visible to the different user communities and at the same time give credit to the researchers that produce information and knowledge that have a value inside *and* outside academia.

Biodiversity researchers were slow to embrace the Web and use digital data but this is history [10]. Today several major initiatives are running to digitize biological collections, library stocks, and are linking collection databases on a global scale (e.g. Biodiversity Heritage Library, Global Biodiversity Information Facility, Encyclopedia of Life, Species2000, ViBRANT). The Web is adopted not only by organizations, but by individual researchers too, who have started to collaborate online in so called “virtual research communities” using web-based e-infrastructures, such as Scratchpads, the case presented in this paper.

Scratchpads are online collaboratories (smart websites) for experts and other interested users in biodiversity research. They facilitate sharing, storing and analyzing biodiversity data. Scratchpad owners can choose if they make all or only part of the content of their website publicly available. Visitors to the Scratchpad sites can be either registered users or anonymous visitors.

**References:**

1. Krell FT (2000) Impact factors aren't relevant to taxonomy. Nature, 405, 6786: 507-508. doi:10.1038/35014664.

2. Krell FT (2002) Why impact factors don't work for taxonomy. Nature, 415, 6875: 957. doi: 10.1038/415957a.

3. Agnarsson I, Kuntner M (2007) Taxonomy in a changing world. Seeking solutions for a science in crisis. Systematic Biology 56, 3: 531-539. doi: 10.1080/10635150701424546.

4. Godfray HCJ (2002) Challenges for taxonomy. Nature, 417,6884: 17-9. doi: 10.1038/417017a.

5. Valdecasas AG, Castroviejo S, Marcus LF (2000) Reliance on the citation index undermines the study of biodiversity. Nature 403: 698. doi: 10.1038/35001751.

6. Wheeler QD, Valdecasas AG (2005) Ten challenges to transform taxonomy. Graellsia 61,2: 151-160. http://graellsia.revistas.csic.es/index.php/graellsia/article/download/17/17. Accessed February 21 2012.

7. Werner YL (2006) The case of impact factor versus taxonomy: a proposal. Journal of Natural History, 40, 21-22: 1285-1286. http://dx.doi.org/10.1080/00222930600903660.

8. House of Lords' Select Committee on Science and Technology (2009) Systematics and Taxonomy Follow-up: Government Response. 1st report (2008-9) (HL Paper 58). London: The Stationery Office Limited. 24 p.

9. Graham C, Ferrier S, Huettman F, Moritz C, Peterson A (2004) New developments in museum-based informatics and applications in biodiversity analysis. Trends in Ecology & Evolution 19, 9:497-503. http://dx.doi.org/10.1016/j.tree.2004.07.006.

10. Penev L, Roberts D, Smith, VS, Agosti D, Erwin, T (2010) Taxonomy shifts up a gear: New publishing tools to accelerate biodiversity research. ZooKeys 50, i-iv. doi: 10.3897/zookeys.50.543.
