# Supplementary material for: Identifying Audiences of E-Infrastructures - Tools for Measuring Impact
Source: PLoS One. 2012 Dec 11;7(12):e50943. doi: 10.1371/journal.pone.0050943 (PMC3519820; doi:10.1371/journal.pone.0050943)
Supplement: Text S2 — Development of the two filter sets. This describes in more detail the development of the two filter sets. (DOCX) [file pone.0050943.s002.docx]

*Supplementary information S2*

*Development of the two filter sets*

We produced two filters sets, one the product of a lengthy, manual review of the initial data in Google Analytics comprises of 181 terms, the second is a far more quickly prepared, machine learning assisted set comprising of 6 terms. We used our gold standard manually coded data to compare the two filter sets.

To apply the filters to our test data we wrote a small script that applies the same pattern matching technique as Google Analytics. However, a benefit of writing our own tool was that we could enhance it to better meet our needs, specifically in how we could review the results.

The primary output from the script is a marked up copy of the gold standard data, a new column being added alongside the hand-marked column. This is shown in the screenshot in Figure S1. Column D contains the hand marked identification of that ISP, and column E contains the identification given by the script. Using Excel’s auto filter functionality, it is easy to isolate mismatches between the two columns, which greatly aids refinement of the filters.


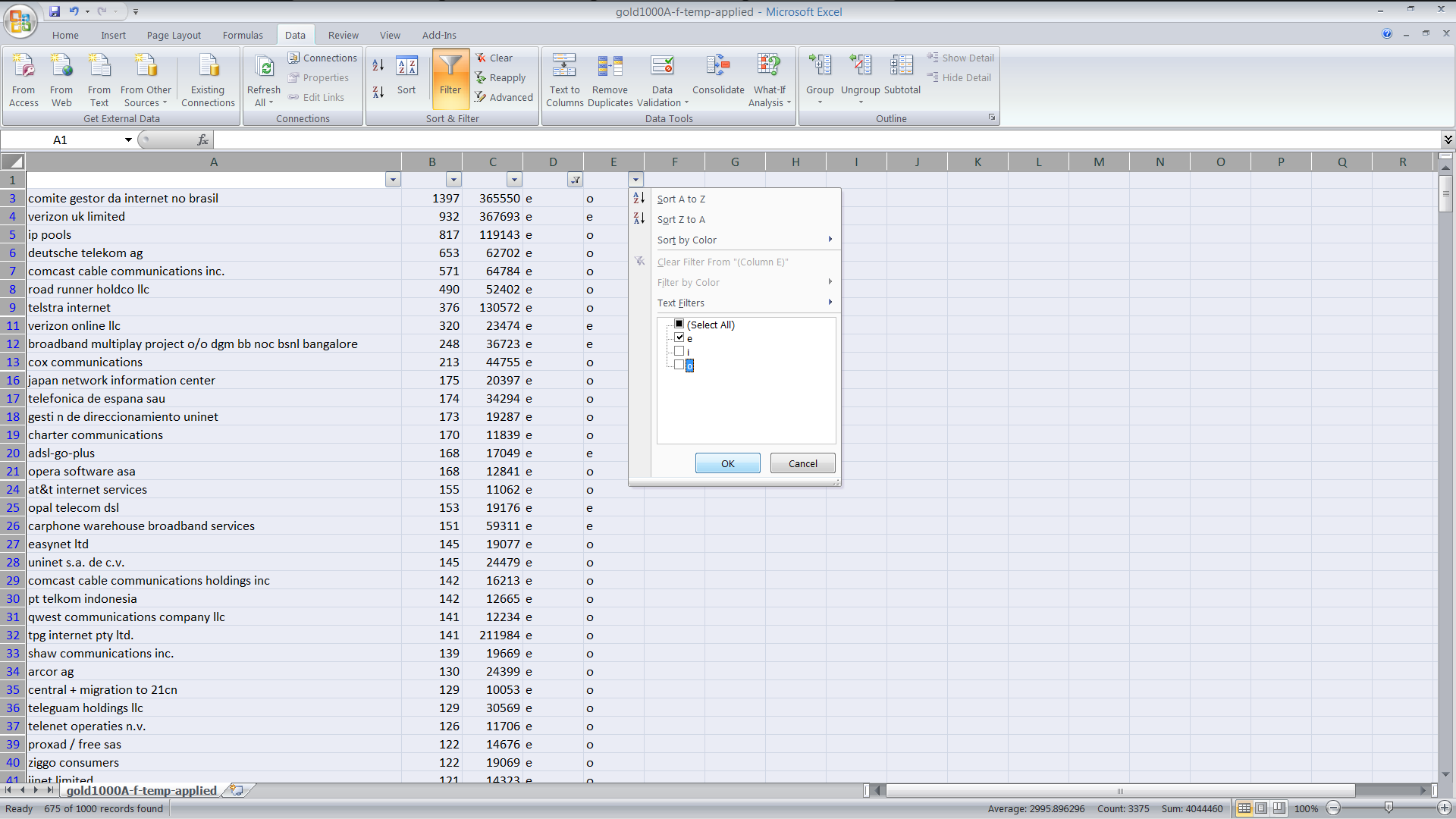


**Figure S1: Screenshot of filtered output.**

This figure shows the use of Excel to aid review of our filter sets

through quickly identifying matches and mismatches in the gold

standard test data output.

Our script produces logs for all of its actions, which serve both to confirm which identification was made, and that if no identification can be made that the default is applied:

found natur in natural history museum

found univer in freie universitaet berlin

nothing found in usda office of operations ** marked as other **

This is useful on two accounts. Firstly, we can see which term in the filter set identified the VO, which is very useful when resolving incorrect matches. Secondly, by isolating the ‘nothing found’ entries, we can examine which VOs the filter missed, and therefore what enhancements might be made to the filter sets.

The following tables demonstrate the improvements we were able to make to the filter sets following review of the script’s output. All results we present relate to our full gold standard test data set of which 303 entries have been hand marked as include, 674 exclude and 23 other, for a total of 1,000 entries. Our first test was to apply the filter set manually derived from review of the Google Analytics data to the gold standard test data using the same pattern matching algorithm as Google. The results are shown in Table S1.

**Table S1: Results applying the manual filter set to gold standard test data.**

|  | **include** | **exclude** | **Other** |
| --- | --- | --- | --- |
| false positive | 31 | 1 | 537 |
| false negative | 12 | 549 | 8 |
| true positive | 290 | 126 | 15 |
| true negative | 667 | 324 | 440 |
| precision | 0.90 | 0.99 | 0.03 |
| recall | 0.96 | 0.19 | 0.65 |
| f-measure | 0.92 | 0.32 | 0.06 |

On reviewing the logs we could immediately see that many incorrectly identified *include* ISPs were being matched by the term “gmbh”. Removal of this one term from the ‘include’ filter set produces the results in Table S2.

**Table S2: Results applying manual filter set to gold standard test data after removal of “gmbh” from the include list.**

|  | **include** | **exclude** | **other** |
| --- | --- | --- | --- |
| false positive | 19 | 1 | 546 |
| false negative | 12 | 547 | 7 |
| true positive | 290 | 128 | 16 |
| true negative | 679 | 324 | 431 |
| precision | 0.94 | 0.99 | 0.03 |
| recall | 0.96 | 0.19 | 0.70 |
| f-measure | 0.94 | 0.32 | 0.06 |

Having achieved good precision and recall measures for the VOs to include, we now reviewed the logs to see why so few of ISPs to exclude were identified, as shown by the low recall measure of 0.19. This manual review of the output from applying filter-sets to the gold standard data suggested that the addition of two terms, “internet” and “verizon”, should improve the overall accuracy of classification. Adding these two terms does indeed show a marked improvement in performance of the exclude filter, without making the performance of the include or the other filter worse as shown in Table S3.

**Table S3: Results applying manual filter set to gold standard test data after addition of “internet” and “verizon” to the exclude list.**

|  | **include** | **exclude** | **other** |
| --- | --- | --- | --- |
| false positive | 19 | 1 | 507 |
| false negative | 12 | 508 | 7 |
| true positive | 290 | 167 | 16 |
| true negative | 679 | 324 | 470 |
| Precision | 0.94 | 0.99 | 0.03 |
| Recall | 0.96 | 0.25 | 0.70 |
| f-measure | 0.94 | 0.40 | 0.06 |

Continuing detailed manual review of the results and output proved to us the value of this approach, and the benefit of developing our own tools because we could easily assess individual changes.
